# Desafíos actuales de la neurología clínica en el diagnóstico de las enfermedades neurodegenerativas

**DOI:** 10.7705/biomedica.8336

**Published:** 2026-03-02

**Authors:** Rodrigo Pardo-Turriago

**Affiliations:** 1 Profesor titular; coordinador, Unidad de Neurología, Facultad de Medicina, Universidad Nacional de Colombia, Bogotá, D. C., Colombia Universidad Nacional de Colombia Facultad de Medicina Universidad Nacional de Colombia Bogotá D. C Colombia

El excitante progreso de la biología molecular y de las ciencias ómicas ha creado nuevas perspectivas para la práctica neurológica frente a las llamadas enfermedades neurodegenerativas.

No resulta del todo claro cuándo ni quién introdujo el concepto de «enfermedad neurodegenerativa» en la práctica clínica, pero es indudable que la atención que hacia la mitad del siglo veinte suscitaban la enfermedad de Alzheimer y la de Parkinson, entre otras, permitió abrir un nuevo capítulo en los textos de Neurología y Neuropatología, para dar cabida a una serie de afecciones del sistema nervioso cuya etiología y fisiopatología parecían tanto sorprendentes como enigmáticas.

El ejercicio de la Neurología, influenciada por la fascinación que ejercen la semiología y la fenomenología entre sus practicantes, se dedicó a la descripción cuidadosa de los fenotipos y sus variantes, a la identificación de aquellos rasgos comunes o diversos que permitieron las primeras clasificaciones y la formulación de hipótesis sobre sus causas, y a su agrupación bajo diferentes criterios.

Vale la pena recordar que, si bien en la primera mitad del siglo XX fueron las infecciones, en particular la sífilis y la tuberculosis, los marcos de referencia para explicar algunas de las enfermedades neurológicas crónicas -arrebatando a la enfermedad mental su preeminencia, como en el caso de la parálisis general progresiva o la tabes dorsal-, muy pronto sería la aterosclerosis el mecanismo fisiopatológico que se invocaría como el responsable de la demencia entonces llamada «senil» y, que de allí surgió el uso extendido de medicamentos contra la arteriosclerosis como la forma expedita de resolverla. ¡Qué paradoja cuando hoy aceptamos a la arteriosclerosis como un proceso degenerativo mediado por intrincados procesos bioquímicos y moleculares!

Pero, antes que resolver la inquietud y la curiosidad, se reconocía la dificultad para entender cabalmente un sinnúmero de condiciones que afectan al sistema nervioso y se caracterizan por un inicio en la edad adulta, un curso progresivo, una acumulación de daño tisular y un compromiso funcional que conduce hacia la pérdida de la capacidad funcional y de autonomía.

Surgen, entonces, las explicaciones basadas en la citotoxicidad, en especial debido al influjo del calcio en exceso, por alteración funcional de los canales iónicos de membrana y la señalización intracelular. Los mecanismos de oxidación anormal ofrecían una vía conveniente para explicar los cambios de pérdida neuronal, falla sináptica y necrosis tisular.

Entre tanto, la práctica clínica se esforzaba en el examen cuidadoso de las formas de la enfermedad y sus manifestaciones, reforzando la fascinación que produce en el neurólogo asomarse a la increíble perfección de un sistema y a la perturbación de su funcionamiento.

¿Cuál es, a su vez, la perspectiva del paciente y su familia, enfrentados a un futuro poco prometedor y de desesperanza? La enfermedad neurodegenerativa se encarna como estigma y factor de exclusión, salvo por la mirada compasiva de quien acompaña. Esto explica una gran proporción de la carga global de la enfermedad en el mundo.

Varios hechos marcaron una importante inflexión en este camino, auspiciados en las últimas décadas del siglo XX, pero especialmente en los últimos veinte años, por el avance de la biología molecular y de la genética.

El entender los mecanismos moleculares que subyacen a la neurodegeneración ha tenido un real impacto en la capacidad de clasificar las enfermedades degenerativas y orientar hacia la búsqueda de soluciones basadas en mecanismos mejor conocidos.

Se impone un cambio de mentalidad y de nomenclatura bajo el concepto de enfermedades conformacionales -o proteinopatías- y, de esta manera, aceptar que los cambios sutiles en la estructura de las proteínas traen consigo interacciones moleculares, agregación y daño tisular.

Por este camino, hemos aprendido que es la agregación de proteínas el fenómeno biológico que juega un papel central en la neurodegeneración. Pero tras ella, quizá sea la traducción defectuosa del ARN mensajero el secreto presente en muchos casos, como se reconoce hoy en la enfermedad de Alzheimer, la demencia fronto-temporal, la enfermedad de Parkinson, la enfermedad de Huntington, las enfermedades atribuidas a priones, la esclerosis lateral amiotrófica, la atrofia muscular espinal, la neuropatía de Charcot-Marie-Tooth y las variantes de la ataxia espinocerebelosa, entre otras.

Desde otra perspectiva, está el impulso de la genética y su indeclinable compromiso de explicar la variación biológica como resultado de la mutación y la reorganización de las proteínas generada por errores en los aminoácidos codificados a partir de cuatro nucleótidos con una innumerable capacidad de combinación que da lugar a familias que comparten rasgos de enfermedades neurodegenerativas. Ello ha permitido una nueva forma de concebir el problema. Las mutaciones genéticas identificadas en las formas familiares han sido el punto de partida para entender el proceso patológico subyacente en los modelos biológicos; en particular, el estudio de las mutaciones dominantes que conducen a la agregación de proteínas, con la consecuencia de un efecto tóxico sobre la función. Las enfermedades neurodegenerativas pueden provenir de mutaciones que afectan la actividad proteica, en mayor proporción que de la pérdida de una proteína activa.

En este marco de referencia, podemos entender la enfermedad neurodegenerativa en su interesante variedad fenomenológica, como la expresión de mecanismos patogénicos comunes: proteínas con alteraciones conformacionales que facilitan interacciones moleculares patógenas que conllevan a su agregación y depósito, produciendo daño tisular. Con qué inmensa profundidad lo explicaba el profesor Francisco Lopera cuando afirmaba que la neurodegeneración no era otra cosa que el efecto del «mugre» que se acumulaba en el tejido nervioso hasta lesionarlo irremediablemente.

Varias preguntas deben aún resolverse: ¿cómo explicar la especificidad regional para cada enfermedad?, ¿cómo explicar en cada caso los mecanismos de toxicidad y las variaciones de la proteína implicada (monómeros, protofibrillas, fibrillas, agregados)? y ¿por qué la expresión de estas proteínas anormales parece estar en función de la edad?

En este contexto, la lista de enfermedades neurodegenerativas continuará creciendo y los neurólogos clínicos tendremos que aprender a mirar este fenómeno de otra manera. Leeremos en los textos modernos capítulos sobre proteinopatías, como las angiopatías amiloides cerebrales, las taupatías, las sinucleinopatías y las expansiones de repeticiones de poliglutamina, entre otras. Su agrupamiento ya no dependerá exclusivamente de sus manifestaciones clínicas o de los efectos que producen o de los sistemas afectados. En su lugar, los mecanismos biológicos implicados jugarán un papel destacado.

No abandonaremos los magníficos recursos de una práctica clínica sólida y rigurosa, de hacer visible lo invisible, del uso correcto del oftalmoscopio y del martillo de reflejos, y de la valoración del tono o de la fuerza. Por años, estos instrumentos utilizados con un criterio estricto anclado en una morfología exquisita y una fisiología viva nos han permitido adentrarnos en el sistema nervioso y su enigmática majestad. Con ellos, resulta posible examinar los distintos sistemas del neuroeje y de sus proyecciones periféricas, establecer el daño, su naturaleza y su topografía, calificar el impacto e imaginar el pronóstico.

Pero a todas luces, no será suficiente. Seguir el paso al avance de los descubrimientos que nos brindan las llamadas "ciencias básicas" es una tarea inaplazable. El entender los mecanismos moleculares, celulares, bioquímicos, genéticos y de las ciencias ómicas implicadas en la fisiopatología de la enfermedad neurodegenerativa, será tan importante como reconocer su presencia y evaluar su impacto. Es, sin duda, un nuevo reto para el neurólogo de hoy, de quien se reclama una profunda formación clínica, y un ejercicio compasivo y empático frente a la enfermedad neurodegenerativa.

No se trata de cambiar nuestra práctica. Se trata de expandir sus confines.

Es una nueva epistemología, una nueva forma de conocer, entender, practicar y comunicar la neurología. Implica abarcar la enfermedad neurodegenerativa con una mirada más amplia, y aceptar nuestra ignorancia y nuestras limitaciones.

Basta con revisar los índices temáticos de las publicaciones periódicas que a diario acostumbramos a leer, para advertir la realidad que ellos retratan. La investigación básica adquiere un vertiginoso ritmo y, a pesar de nuestro esfuerzo, es difícil seguirle el paso. La innovación, la tecnología, la simulación y la inteligencia artificial nos brindan una enorme cantidad de información que nos obliga a ser lectores competentes, críticos y racionales. La emergencia de biomarcadores para identificar las proteínas anormales comprometidas, sus daños conformacionales o sus consecuencias están a la orden del día y sus datos circulan cómodamente en las redes informáticas que consumen nuestros pacientes, quienes esperan una respuesta a sus múltiples inquietudes, cuando no es que nos sorprenden con un mayor conocimiento que el de quien debe orientarlos.

Todo ello implicará introducir cambios curriculares en los programas de entrenamiento, fortalecer la comunicación interdisciplinaria, abrir nuevos espacios de práctica, incorporar los resultados de la innovación tras un ejercicio de aproximación crítica reflexiva y ponderada, y regresar a los conceptos básicos y avanzados en genética, biología molecular y bioquímica, sin olvidar el gran acervo científico de nuestra especialidad.

El diagnóstico de una enfermedad neurodegenerativa coloca al clínico frente al dilema ético de descubrirla y no poder ofrecer una solución que permita reversar el proceso patológico en curso. Es la frustración del presente y la esperanza del futuro. El vigor con el que se desarrolla la investigación sobre nuevas moléculas dirigidas a resolver las interacciones moleculares mencionadas nos invita a ser optimistas y a desarrollar las capacidades requeridas. La historia del cáncer es un buen referente.

Por último, nuestro mayor valor es, sin duda, la aproximación empática, la atenta escucha, la capacidad de entender y de acompañar al paciente afectado y a su familia, el honrar la inmensa herencia de Philippe Pinel y su acumulado histórico. El gran reto para el neurólogo en este nuevo contexto es validar su ejercicio profesional, incorporando a su práctica un nuevo conocimiento, nuevas opciones para entender, explicar y acompañar y, como siempre, seguir su deber hipocrático: nunca hacer daño. ¡Bienvenido el reto!

